# Exosomes and osteosarcoma drug resistance

**DOI:** 10.3389/fonc.2023.1133726

**Published:** 2023-03-17

**Authors:** Huichao Fu, Yunjiao Wu, Jianbai Chen, Xing Hu, Xiaoyan Wang, Gongping Xu

**Affiliations:** ^1^ Department of Orthopedic Surgery, The Second Affiliated Hospital of Harbin Medical University, Harbin, Heilongjiang, China; ^2^ Department of Respiratory Medical Oncology, Harbin Medical University Cancer Hospital, Harbin, Heilongjiang, China

**Keywords:** osteosarcoma, exosomes, biomarkers, drug resistance, treatment

## Abstract

Osteosarcoma (OS) is a primary malignant tumor of bone characterized by the formation of bone tissue or immature bone by tumor cells. Because of its multi-drug resistance, even with the improvement of chemotherapy and the use of targeted drugs, the survival rate of osteosarcoma (OS) is still less than 60%, and it is easy to metastasize, which is a difficulty for many clinicians and researchers. In recent years, with the continuous research on exosomes, it has been found that exosomes play a role in the diagnosis, treatment and chemotherapy resistance of osteosarcoma due to their unique properties. Exosomes can reduce the intracellular accumulation of chemotherapeutic drugs by mediating drug efflux, thus inducing chemotherapeutic resistance in OS cells. Exosomal goods (including miRNA and functional proteins) carried by exosomes also show great potential in affecting the drug resistance of OS. In addition, miRNA carried by exosomes and exosomes exist widely in tumor cells and can reflect the characteristics of parent cells, so it can also be used as a biomarker of OS. At the same time, the development of nanomedicine has given a new hope for the treatment of OS. Exosomes are regarded as good natural nano-carriers by researchers because of their excellent targeted transport capacity and low toxicity, which will play an important role in the field of OS therapy in the future. This paper reviews the internal relationship between exosomes and OS chemotherapy resistance, discusses the broad prospects of exosomes in the field of diagnosis and treatment of OS, and puts forward some suggestions for the study of the mechanism of OS chemotherapy resistance.

## Introduction

Osteosarcoma (OS) is the most common primary malignant bone tumor originating from mesenchymal cell (1, 2). Most OS cases are characterized by bimodal distribution of age. Most cases occur in adolescents aged 10-14 years, followed by people over 60 years old (3). It is the most common in the metaphysis of long bone, and the three most common sites are distal femur, proximal tibia and proximal humerus (4). It is well known that radiotherapy is rarely used in the treatment of OS because of the radiation resistance of OS. At present, OS patients are treated with a multidisciplinary approach established by Rosen. et al. in the 1970s (5), that is, chemotherapy (neoadjuvant and adjuvant, lasting 6 to 8 months) combined with surgical treatment, the 5-year survival rate of patients can reach 70% (6–8). However, due to the resistance of OS cells to chemotherapeutic drugs and the lack of biomarkers to predict treatment response. In the past 40 years, limited progress has been made in improving the survival outcome of patients with OS (9). Therefore, it is necessary to further understand the potential mechanism of drug resistance, to find reliable biomarkers that can predict treatment response and to explore new treatments for the treatment of OS and to improve the survival rate of patients (10).

**Figure 1:**
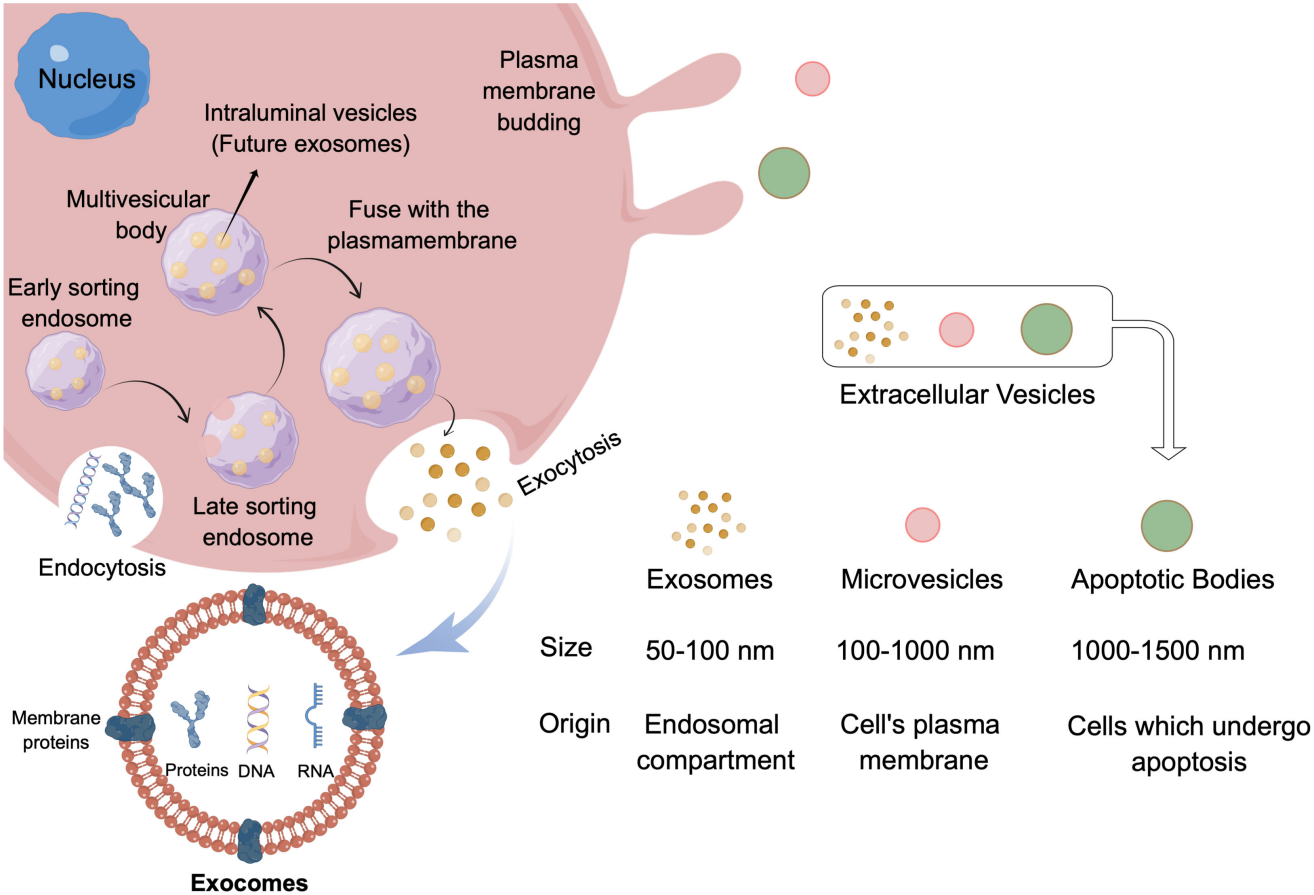
Classification of extracellular vesicles and exosomes biogenesis and structure. EV is divided into small EV (50-100 nm), medium EV (100-1000 nm) and large EV (1000-1500 nm) according to its size, and according to its origin can divide into exosomes (endosomal compartment), microvesicles (cell’s plasma membrane) and apoptotic bodies (cells which undergo apoptosis). Exosomes originated from the endogenous pathway, through the formation of ESEs, LSEs, and finally formed MVBs, which contains ILVs. When MVBS fuses with the cell membrane, the exosome is released. By Figdraw.

Extracellular vesicles (EVs) are small lipid-binding vesicles released by cells carrying heterogeneous items (including proteins, RNA, miRNA, other non-coding RNA, DNA, lipids and metabolites). This kind of goods can be transferred to other cells and can affect their physiological functions, thus playing an important role in cell-to-cell communication. EVs are divided into small EV (50-100 nm), medium EV (100-1000 nm) and large EV (1000-1500 nm) according to its size, and exosomes (endosomal origin), micro vesicle (plasma membrane budding) and apoptotic body according to its origin/biogenesis (11) (**Figure 1**). The exosomes belong to the small EV group and the single membrane vesicles which have the same topological structure as the cells (12). The production of exosomes is a complex process, which involves the double invagination of cell membrane and the formation of intracellular multivesicular bodies (MVBs) containing intracavitary vesicles (ILVs) (11) (**Figure 1**). Different intracellular regulatory processes produce different exosomes and give them specific substances, resulting in different biological function (13). The exosomes transfer these substances to the recipient cells to complete intercellular communication. Studies have shown that almost all mammalian cells can secrete and absorb exosomes (14, 15), even tumor cells produce more exosomes than normal cells, and tumor-derived exosomes have a stronger ability to change the microenvironment (16). Based on the available evidence it is not difficult to find that exosomes, especially originate from cancer cells, play an important role in mediating drug resistance, and it also shows its broad prospect in tumor treatment and diagnosis (17).

Garimella. et al. first reported the function of OS cell-derived exosomes, proposing that exosomes drive osteoclast bone resorption by disrupting the dynamic balance of bone reconstruction in the OS bone microenvironment (BME) (18). Existing studies suggest that exosomes derive from OS cells play a crucial role in tumorigenesis, appreciation, metastasis, anti-apoptosis, immune evasion and chemoresistance (19). This article reviews the latest progress of exosomes derived from OS and OS drug resistance, and puts forward suggestions for future studies of exosomes and OS drug resistance on the basis of the mechanism of exosomes affecting other tumor drug resistance. It also discusses the potential role of exosomes as tumor biomarkers in predicting treatment response and its application in anti-tumor drug resistance therapy.

## Exosome and chemoresistance in osteosarcoma

According to the former study, preoperative and postoperative chemotherapy regimens based on different combinations of doxorubicin, methotrexate, and cisplatin with the possible addition of ifosfamide, etoposide and, more recently, liposomal muramyl tripeptide phosphatidylethanolamine constitute the common treatment for primary conventional high-grade osteosarcoma (HGOS) (20–24). However, osteosarcoma (OS) can avoid chemotherapeutic drug-induced cell death through a variety of mechanisms, including: reduction of intracellular drug accumulation, drug inactivation, improvement of DNA repair, regulation of signaling pathways, autophagy-related drug resistance, turbulence in cell cycle-related gene expression, and even microenvironmental effects (25). In addition, recent studies have reported the discovery of exosomes in the development of OS drug resistance. It is involved in mediating drug resistance in OS, including exosome-mediated drug efflux, exosome shuttle RNA-mediated drug resistance, exosomal cargo-mediated drug resistance. In this review, we summarize the latest progress on the association of exosomes with OS drug resistance (**Table 1**).

**Table 1:** Mechanisms underlying OS drug resistance mediated by exosomes.

Cell origin of exosome	Exosome content	Target(s)	Resistant type	Mechanism	Reference
DOX-resistant cells	Chemotherapeutic drug	Promoting drug efflux	DOX resistance	DOX can be excreted through exosomes	(26)
DOX-resistant cells	P-gp	Promoting drug efflux	DOX resistance	Transferring drug resistance as well as P-gp from drug-resistant OS cells to sensitive ones	(27)
DOX-resistant cells	MDR-1 mRNA	P-gp	DOX resistance	In sensitive cells, encodes p-gp and promotes drug efflux from sensitive cells	(27)
HGOS cells	miR-25-3p	DKK3	Unknown	Promotes tumor growth, invasion and drug resistance	(28)
DOX-resistant cells	lncRNA ANCR	Unknown	DOX resistance	Promotes tumor growth, invasion and drug resistance	(29)
CDDP-resistant cells	HSA_CIRC_103801	Unknown	CDDP resistance	Reduced cellular sensitivity to CDDP and increased expression of multidrug resistance-associated protein 1 and P-gp	(30)

DOX, doxorubicin; CDDP, cisplatin; HGOS, high-grade osteosarcoma; MDR-1, multidrug resistance-1; DKK3, dickkopf WNT signal pathway inhibitor 3; P-gp, permeability glycoprotein.

### Exosomes-mediated drug efflux

**Figure 2:**
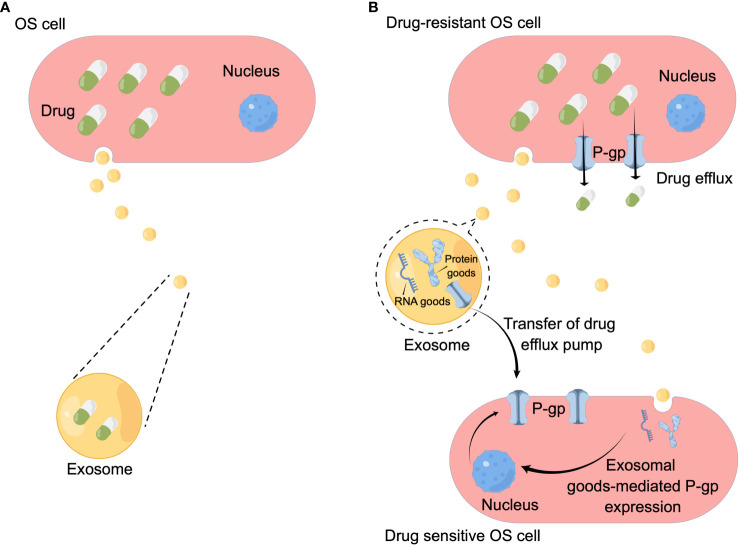
Mechanisms underlying OS drug resistance mediated by exosomes. **(A)** Chemotherapeutic drugs are secreted when they are encapsulated in the exosomes. **(B)** Exosomes mediate horizontal transfer of membrane-embedded drug efflux pumps to sensitive cancer cells to favor the drug efflux. Exosomes also deliver functional proteins/miRNAs to upregulate P-gp expression in sensitive cancer cells. By Figdraw.

In tumor cells, the full accumulation of chemotherapeutic drugs is a prerequisite for its curative effect, so increasing drug efflux is an important mechanism leading to chemotherapy resistance. Studies have shown that there are at least three main mechanisms of exosomes-mediated drug efflux in OS: the first is exosomes-mediated drug efflux. In 2003, Kerby Shedden et al. proved that the expression of genes related to vesicle exfoliation in tumor cells was positively correlated with their drug resistance, and they found that tumor cells could excrete the chemotherapeutic drug doxorubicin through vesicles (26) (**Figure 2**). The second mechanism is that exosomes mediate OS resistance by mediating the horizontal transfer of drug efflux pumps to sensitive cancer cells (**Figure 2**). Adenosine triphosphate (ATP) binding cassette (ABC) transporter uses ATP to excrete various exogenous substances, including anticancer drugs with different structures and properties (31, 32). ABC subfamily B member 1 gene encodes a drug transporter protein, permeability glycoprotein (P-gp) (33). Numerous studies have shown that P-gp can be transferred from drug-resistant tumor cells to sensitive cells *via* exosomes, leading to acquired drug resistance in drug-sensitive cells (34–36). Data from the study by Torreggiani et al. showed that exosomes derived from doxorubicin-resistant OS cells could be taken up into secondary cells and induce a doxorubicin-resistant phenotype (27). They demonstrated that P-gp is contained in drug-resistant cell derived-exosomes and that multidrug resistance (MDR) can be transferred to sensitive cells by the delivery of P-gp in recipient cells through exosomes. Meanwhile the same authors found that the presence of MDR-1 mRNA (encoding ABCB1), besides P-gp, was detected in exosomes produced by drug-resistant OS cells, which also suggests that, in addition to mediating OS drug resistance by transporting the drug efflux pump P-gp, exosomal transport of resistance-related coding RNAs is also a mechanism leading to OS drug resistance (27). These results suggest that exosomes play an important role in the drug resistance process in OS by directly exporting chemotherapeutic drugs, mediating the delivery of drug efflux pumps, and transporting RNAs associated with drug resistance.

On the other hand, investigators found that in drug-resistant OS cells, exosomes upregulate P-gp expression and thus mediate drug resistance by mediating the transfer of transient receptor potential channels (TRPCs) (37–39). In 2017 Ning et al. found that ubiquitin carboxy-terminal hydrolysate-L1 (UCH-L1) of exosomes can upregulate P-gp expression by activating the mitogen-activated protein kinase/extracellular regulated protein kinase signaling pathway to upregulate the expression of P-gp (40). Taken together, these studies demonstrate that OS-derived exosomes can also regulate P-gp expression and thus mediate BC drug resistance by mediating the transfer of functional proteins, but this mechanism has not been confirmed in OS cells. Therefore, the authors believe that this would be a new direction to explore the mechanism of exosome-mediated OS drug resistance.

### Exosome shuttle RNA mediates drug resistance in OS cells

In the 2010s, researchers demonstrated that miRNA and mRNA can be loaded as “goods” in exosomes. The exosome carries RNA that can shuttle between cells, so it is called “exosome shuttle RNA”. As more and more studies have found that exosome shuttle RNA plays an important role in intercellular communication (41), researchers have begun to explore the relationship between exosomal RNA and drug resistance. Research have confirmed RNA in exosomes produced by tumor cells can mediate tumor drug resistance (42, 43) (**Figure 2**), and this mechanism also exists in OS cells. Human HGOS tissues had an abnormal miR-25-3p expression which inhibited the expression of dickkopf WNT signal pathway inhibitor 3 (DKK3) gene, according to an article from 2018 (28), and was found to be negatively correlated with clinical outcomes. The same authors demonstrated that in the HGOS experimental model, up-regulation of miR-25-3p promoted tumor growth, invasion and drug resistance, and the same effect was detected after DKK3 silencing. An interesting finding was that tumor-derived exosomes contain miR-25-3p, suggesting that it may play its carcinogenic role through exosomes-mediated transmission. At the same time Xin Hu et al. (29) had reported exosomal long non-coding RNA ANCR mediates drug resistance in OS. They found exosomes derived from doxycycline (dox)-resistant cells can drive *in vitro* chemoresistance to dox in dox-sensitive cells, and the expression of DANCER (encoding lncRNA) in exosomes of dox-resistant cells was higher than that in exosomes of dox-sensitive cells, testified the high expression of the lncRNA ANCR is related to dox-resistant in OS. Another study from the same year (30) noted that exosomes, originated from cisplatin-resistant OS cells, confer cisplatin resistance to recipient cells in an exosomal circ_103801-dependent manner. Circular RNAs (circRNAs) are a kind of non-coding RNAs that are formed by back-splicing events. The expression of HSA_CIRC_103801, a novel discovered circRNA, was shown to be upregulated in OS tissues and cell lines (44). Researchers first reported the existence of exosome-derived HSA_CIRC_103801 in cisplatin (CDDP)-resistant OS cells, and proved that exosome-derived HSA_CIRC_103801 produced by CDDP-resistant cells can enhance the resistance of OS cells to CDDP (30). These results suggest that exosomes derived from drug-resistant OS cells can mediate drug resistance of chemotherapy-sensitive OS cells by carrying RNA with different function.

In addition, with deep investigations of miRNA in recent years, a host of studies have validated miRNAs are involved in the formation of OS drug resistance (45). As oncogenic or tumor suppressor miRNAs, these miRNAs play an important role in chemosensitivity by increasing DDR, preventing apoptosis, triggering autophagy, and activating CSCs (46). However, only three of the above-mentioned non-coding RNAs have been confirmed and reported to mediate OS drug resistance by exosomes as vectors. Therefore, it is necessary to perform compositional analysis of exosomal cargoes in subsequent studies of OS to search for non-coding RNAs and thus explore their relationship with drug resistance.

### Exosomal cargo-mediated drug resistance

Exosomes are functional protein and small RNA containing extracellular vesicles that are able to mediate hetero-and homotypic intercellular communication (47). This means that in addition to mRNA, the functional proteins carried by exosomes can also mediate drug resistance in OS cells (**Figure 2**). In a recent study, exosomal protein cargo was studied using an experimental model of canine OS and clinical samples (48). In this study, the protein content of exosomes derived from two kinds of canine OS and carboplatin-resistant variants (HMPOS-2.5R and HMPOS-10R) was compared with that of their drug-sensitive parent cell line HMPOS. The authors demonstrated that exosomes exhibit different protein characteristics associated with drug resistance, and exosomes from drug-resistant HMPOS-2.5R variants can transfer drug resistance to drug-sensitive HMPOS cells. At the same time, researchers have found the expression of β-catenin was upregulated in the carboplatin resistant cell lines, β-catenin can induce drug resistance by various mechanisms, including upregulation of MDR1 and promotion of epithelial-mesenchymal transformation. Despite the β-catenin protein was not detected in the exosomal cargo, they have confirmed signal transduction of this pathway and speculated that exosomes of resistant cells may promote the expression and phosphorylation of β-catenin in naive cells (48). The above results demonstrate that there is a link between OS-derived exosomal proteins and OS drug resistance, but the exact mechanism still needs further study in the future.

## Exosomes and diagnosis in osteosarcoma

Tumor biomarkers can predict response to specific treatments and may reveal tumor sensitivity or resistance to drugs. Therefore, liquid biopsy analysis (circulating tumor cells, cell-free DNA, exosomes, and proteins) has received a lot of attention from researchers because of its minimally invasive and reproducible nature (49–51). As we gain an understanding of exosome biology and its role in tumor therapy resistance, due to their unique features (carrying surface markers, their cargos reflect the physiological state of the cell they originated from, relatively stable structure, and presence in almost all biological fluids), exosomes can be used as biomarkers of response or resistance to therapy and as early indicators of disease progression (52–54). In this review, we summarize published clinical studies on exploiting exosomes as diagnostic, prognostic, or predictive biomarkers in OS (**Table 2**).

**Table 2:** Published clinical studies on exploiting exosomes as diagnostic, prognostic, or predictive biomarkers in OS.

Clinical studies	Diagnosis	Prognosis		Target therapy	Reference
Fujiwara T, 2017	Osteosarcoma	AUROC	0.868 (0.743-0.993)	Exosomal miR-25-3p	(55)
		Sensitivity, %	71.4		
		Specificity, %	92.3		
Yoshida A, 2018	Osteosarcoma	Unknown		Exosomal miR-25-3p	(28)
Wang L, 2021	Osteosarcoma	AUROC (1-years DFS)	0.90 (0.83-0.98)	Exosome-derived SENP1	(56)
		Sensitivity, %	87.5		
		Specificity, %	89.1		
		AUROC (3-years DFS)	0.96 (0.94-0.99)		
		Sensitivity, %	91.8		
		Specificity, %	89		
		AUROC (1-years OS)	0.90 (0.82-0.99)		
		Sensitivity, %	85.7		
		Specificity, %	90.6		
		AUROC (3-years OS)	0.96 (0.93-0.98)		
		Sensitivity, %	83.3		
		Specificity, %	95		

SENP1, sentrin sumo-specific protease 1; AUROC, area under the receiver operating characteristic curve; DFS, disease-free survival; OS, overall survival.

Existing studies confirm that transport from tumor cell exosomes can be observed in almost all types of liquid or solid tumors, and some of these studies found that exosomes with differential expression of miRNA and mRNA predict chemotherapy response in OS and can be used as biomarkers of tumor cells (57, 58). At the same time, it has been found that the profiles of miRNA carried by exosomes is a marker of tumor cell type and can reflect the characteristics of parental cells (59, 60), that is to say, miRNA, which originate from the exosomes of OS cells in plasma and reflect the characteristics of OS cells, can also be used as a biomarker of OS patients (61). In the subsequent study, Fujiwara et al. successfully verified that serum exosomal miR-25-3p can be used as a noninvasive blood-based biomarker for tumor monitoring in OS patients (55), meanwhile, a study in 2021 (56) confirmed that the plasma exosome-derived sentrin SUMO-specific protease1 (SENP1) was better than plasma SENP1 as a prognostic biomarker in OS. Exosomes have richer and more complete information than other analyses used in liquid biopsies, providing more accurate results or information for predicting treatment response and monitoring the progression of OS. A recent review (60) summarized the research progress of circRNA as a biomarker for osteosarcoma and mentioned that exosomal circRNA exists in a large number of different sources of body fluids and also carries biological information related to the primary tumor, which can cross various biological barriers and holds great promise for the diagnosis and treatment of osteosarcoma as a biomarker for early diagnosis and prognosis assessment. Presently, two human clinical trials on exosomes as biomarkers of OS are under way, which proves that exosomes have great potential in the field of OS biomarkers (61). But there are still some limitations, for example, standardized methods for collecting, processing, and isolating exosome samples have not been established. Current separation techniques, such as ultracentrifugation, are time consuming and do not allow for high purity separation (62). In addition, how to perform specific identification of tumor-derived exosomes among many different sources of exosomes will also be a challenge for exosomes as OS biomarkers.

## Exosomes and therapy in OS

In OS cells, exosomes can not only induce the transfer of drug resistance from drug-resistant cells to sensitive cells, promote tumor growth and development, but also inhibit tumor progression (65). Due to their unique properties and function, exosomes are regards as a potential treatment of cancers including OS (66). In addition, with the development of nanotechnology and its application in medicine, the therapeutic potential of exosomes has been improved. Despite, chemotherapy is the main auxiliary therapy for OS, systemic injury caused by chemotherapeutic drugs is inevitable. Therefore, it is particularly important to develop a drug delivery system that can directly target OS cells. Researchers have found exosomes are a promising drug delivery system with low immunogenicity, high biocompatibility and high delivery efficiency (67), and have demonstrated the great potential of exosomes as nano-carriers in the treatment of OS (68).

Currently miRNA has been considered as a potential anticancer drug, in a recent study (69), researchers investigated the effect of miR-449a/CCNB1 axis on osteosarcoma through bioinformatics analysis and *in vitro* cellular assays, demonstrating that investigated miR-449a can inhibit osteosarcoma growth. However, because exogenous miRNA is easily degraded in the body, traditional miRNAs delivery methods do not usually achieve the expected effect (62). So, in order to overcome this problem, it is undoubtedly the best choice to choose exosomes as miRNA carriers. Shimbo et al. found that artificial miRNA-143 can be introduced into OS cells through exosomes to play a therapeutic role and reduce bone metastasis. Moreover, they proved the superiority and effectiveness of exosomes as miRNA vectors by comparing with other transfection process (70). At the same time, in another research, Wei et al. developed a new nano drug by combining DOX with exosomes from MSCs. Compared with the effect of free DOX, the new drug has a stronger anti-tumor effect and shows better targeting selectivity, they speculate that it may be attributed to the interaction of the membrane proteins in the surface of MSCs-derived exosomes and OS cells (71). Furthermore, in recent years, studies on EXO membrane proteins have also shown that EXO targeted transport is related to it (72). The results of Han et al. show that the high targeting of exosome dependent delivery system is due to the surface modification of exosome membrane (73). In addition to these, researchers found that the exosomes from mesenchymal stem cells have homing ability similar to that of their parents, and can directly deliver drugs to recipient cells under the condition of protecting them from extracellular degradation (74), and Abello et al. proves this mechanism also exists in OS cells (75). A study in 2022 (76) also demonstrated that exosomes can be used as a safe nanocarrier loaded with miR-665 allowing it to inhibit OS progression *in vivo* and *in vitro* for therapeutic purposes. In another study in the same year (69), Han J et al. concluded that MiR-449a could be used as an anti-cancer agent loaded in engineered exosomes. To sum up, exosomes can play an important role as nanocarriers of drugs in the treatment of OS due to their low cytotoxicity and good target selectivity.

## Drawbacks in the use of exosomes for clinical management

Currently, the great potential of exosomes as diagnostic and prognostic biomarkers for various cancers has been demonstrated, however, to be further used in the clinic, the acquisition of exosomes will be a non-negligible challenge, and despite revolutionary advances in exosome isolation techniques, due to the high heterogeneity and nanometer size of exosomes, there is no standardized method for high-throughput, high purity and minimal damage isolation of exosomes (77). At the same time, it is widely recognized that exosomes can be used as nanocarriers for cancer therapeutics, but how to find exosomes that work specifically among the many exosomes and how to ensure that engineered exosomes exert targeted tumor-killing effects without affecting other cells are challenges that need to be overcome (78).

## Conclusion

Osteosarcoma is clinically considered to be a chemotherapy-resistant tumor, so even with the continuous improvement of chemotherapy and treatment, the survival rate is still not ideal. The existing evidence shows that exosomes, as intercellular communication molecules, play an important role in drug resistance, diagnosis and treatment of OS and have been confirmed by relevant literature. But so far, Food and Drug Administration (FDA) has not approved the use of exosomes as a clinical trial of OS therapy, on the one hand, because the mechanism and specific function of exosomes have not been fully understood, on the other hand, it is necessary to improve the production and storage of exosomes to avoid exosomes inactivation. Generally speaking, OS is still a puzzle to be developed, but the discovery of exosomes and the deepening of its research give us new hope. Therefore, despite many challenges, exosomes may play an important role in the diagnosis and treatment of OS as a biomarker for predicting and monitoring the therapeutic effect of OS, as a drug carrier or as a potential target for reversing drug resistance.

## Author contributions

CHF, YXW, PGX conceived the structure of the manuscript. CHF wrote the manuscript and drew the figures and was a major contributor in writing. JYW, XH, BJC revised the manuscript. All authors contributed to the article and approved the submitted version.
